# Antiperoxidative Activity of *Tetracarpidium conophorum* Leaf Extract in Reproductive Organs of Male Rats

**DOI:** 10.1155/2015/798491

**Published:** 2015-05-07

**Authors:** Seun Funmilola Akomolafe, Ganiyu Oboh, Afolabi Akintunde Akindahunsi, Anthony Jide Afolayan

**Affiliations:** ^1^Department of Biochemistry, Ekiti State University, PMB 5363, Ado Ekiti, Nigeria; ^2^Department of Biochemistry, Federal University of Technology, PMB 704, Akure, Nigeria; ^3^Department of Botany, University of Fort Hare, Alice 5700, South Africa

## Abstract

*Tetracarpidium conophorum* (Mull. Arg.) Hutch. & Dalz is one of the many medicinal plants used in folklore as male fertility enhancers. This research was aimed at evaluating the anti-peroxidative activity of the leaves of this plant by determining their capacity to reduce malondialdehyde (MDA) level in reproductive organs and accessory glands of rats. Adult male rats were administered orally with the aqueous leaf extract from* T. conophorum *at 50, 500 and 1000 mg/kg body weight for 21 consecutive days while clomiphene citrate (1.04 mg/kg body weight), a fertility drug was used as standard. The results of the study indicated that there was increase in relative organ weight, body weight, mean total food and water consumed by the treated groups. Testicular MDA level was highly significantly different from that of the control (*p* < 0.0001) although a tentatively decreased MDA level was observed. However, MDA levels in the reproductive accessory glands, epididymis, seminal vesicle and prostate gland were insignificantly (*p* < 0.05) lower than those of controls. The highest percentage decrease of MDA level (66.35, 42.68, 62.50 and 63.36%) was observed at the highest concentration of the extract (1000 mg/kg) in the testis, epididymis, seminal vesicle and prostate gland respectively. These values were two-fold greater than the values obtained for the standard drug. Interestingly, the treatment of rats with the extract significantly increased the activities of superoxide dismutase, catalase (CAT), glutathione peroxidase (GPx), glutathione-S-transferase (GST) and the levels of GSH, vitamin C and total protein. Collectively, the results suggest that the extract from* T. conophorum *leaves had greater capacity to reduce lipid peroxidation in reproductive organs and accessory glands and thus, this plant may be useful in the treatment/management of reproductive cellular damage involving reactive oxygen species.

## 1. Introduction

Free radical or oxidative damage to sperm is thought to be responsible for many cases of idiopathic oligospermia [1], with high levels of free radicals found in the semen of 40% of infertile men [2]. Carlsen et al. [3] reported that three factors combine to render sperm particularly susceptible to free radical damage, a high membrane concentration of polyunsaturated fatty acids, active generation of free radicals, and a lack of defensive enzymes. All of these factors combine to make the health of the sperm critically dependent upon antioxidants.

Although most free radicals are produced during normal metabolic processes, the environment contributes greatly to the free radical load. Man is exposed to a large number of environmental chemicals [4]. Some are the wastes from industrial and agricultural processes. Some have been designed as structural materials and others have been designed to perform various functions such as healing the sick or killing pests and weeds. Obviously some chemicals are useful but many are toxic and their harm to the environment and our health far outweighs their benefit to society. Men exposed to increased levels of sources of free radicals are much more likely to have abnormal sperm and sperm counts [1, 5].

Sperm are extremely sensitive to free radicals because they are so dependent upon the integrity and fluidity of their cell membrane for proper function. Without proper membrane fluidity, enzymes are activated, which can lead to impaired motility, abnormal structure, loss of viability, and ultimately death to the sperm or teratozoospermia [1, 6]. Moreover, if the oxidative stress increases continuously and the body defense could not cope with it, the problematic male needs more antioxidants. A potential source of effective and safe antioxidants is the medicinal plants.

*Tetracarpidium conophorum* (Mull. Arg.) Hutch. & Dalziel commonly called African walnut is a climbing shrub in the family Euphorbiaceae. The plant is locally cultivated mainly for the nuts which are cooked and consumed as snacks [7]. It is locally used by the elderly people for the treatment of constipation. The amino and fatty acids components of the nut are used for the treatment of prolonged and constant hiccups [8]. The barks are used in coffee as laxative and also chewed to reduce toothache. The leaves, bark, and fruit of the plant are used medicinally and their uses include treatment of giddiness, toothache, eczema, pruritus, psoriasis, common cold, and prostate cancer [9]. Also, in West Africa the leaves are used as male fertility agent and in the treatment of dysentery.* T. conophorum* seeds have been shown to have significant effect (*p* ≤ 0.05) on testosterone and estradiol levels, sperm motility (progressive motile sperm and nonprogressive motile sperm), testes, and epididymides weight of the rats treated with various doses of the seed powder while they increased the level of LH, FSH, sperm viability, and sperm count [10, 11]. In addition, they have been reported to contain several antioxidants that help to promote functions of body systems. Nevertheless, no scientific research has been reported yet on their antioxidant capacity in reproductive system. This research was, thus, aimed at evaluating the antiperoxidative activity of this plant by determining their capacity to reduce malondialdehyde (MDA) level in reproductive organs and accessory glands of treated rats.

## 2. Materials and Methods

### 2.1. Preparation of Plant Extract

Fresh samples of* T. conophorum leaves* were obtained from a farm land near Akure metropolis, Nigeria. Authentication of the sample was carried out at the Department of Plant Science, Ekiti State University, Ado Ekiti, by Mr. Ajayi Ebenezer where voucher specimen (number UHAE 335) was deposited at the herbarium of the same Department. Air dried leaves of the plant were ground into powder. One hundred grams of the ground leaves was then soaked overnight in 1,000 mL of distilled water and filtered through a piece of satin cloth. The filtrate was centrifuged at 5,000 rpm for 15 min and filtered again through Whatman filter paper number 1. The filtrate was freeze dried. The powdered extract was kept at 4°C. The aqueous extract was prepared by dissolving the powder in distilled water to yield concentration of 0.1 g/mL (100 mg/mL) as the stock concentration.

### 2.2. Chemicals

Epinephrine, GSH, 5,5-dithiobis-2-nitrobenzoic acid, hydrogen peroxide, NADP, NADPH, BSA, dithiothreitol, glutathione reductase, trichloroacetic acid (TCA) dinitrophenylhydrazine, thiourea, and thiobarbituric acid (TBA) were purchased from Sigma (St. Louis, MO, USA). All other reagents were of analytical grade and were obtained from the Total Laboratory Technology (Gonubie, South Africa). The standard drug (clomiphene citrate), a fertility drug, was gotten from CIPRA-MEDPRO (PTY) Ltd., Rosen Heights, Pasita Street, Rosen Park, Bellville, 7530, Johannesburg, South Africa.

### 2.3. Experimental Animals

All animal procedures have been approved and prior permission from the University of Fort Hare Animal Ethical Committee was obtained as per the prescribed guidelines. The bioethical allowance reference number was AFO021SAKO01. Twenty-five male albino Wistar rats (8–10 weeks old) weighing between 234 and 327 g were purchased from South African Vaccine Producers (Johannesburg, South Africa) and were housed at the University of Fort Hare Central Animal Unit. The rats were allowed to adapt to the new environment for at least 10 days before the experiment. They were kept under standard condition (inverted 12 h light/dark cycle), constant temperature (22°C ± 2°C), and humidity (70% ± 4%) with excess feeding of water and standard diet (Avi Products (Pty) Ltd. number 21825)* ad libitum*. The animals were handled according to the guidelines of the National Research Council Guide for the Care and Use of Laboratory Animals [12]. Ethical care and handling of experimental animals were observed at all times. Experimental groups were divided with five rats each into 5 groups: Group I served as control and received normal saline, and Groups II, III, and IV received 50, 500, and 1000 mg/kg BW of* T. conophorum* leaf extract orally while Group V served as standard and was given suspension of clomiphene citrate (a fertility drug) (Fertomid-50 tablets, CIPRA-MEDPRO (PTY) LTD) orally at the dose of 1.04 mg/kg BW every day for 21 days. After 21 days of the treatment period, the animals were anesthetized by chloroform and the tissue samples from reproductive organs and accessory glands were collected.

### 2.4. Necropsy

The animals were fasted overnight, weighed, and sacrificed by decapitation 24 h after the last treatment and blood was collected by cardiac puncture. Testes, epididymis, seminal vesicles, and prostate glands were removed and cleared of adhering tissues, washed in ice-cold 1.15% potassium chloride, and dried with blotting paper. The weights of the organs were recorded in gram (g) and also expressed as g/100 g body weight.

### 2.5. Enzyme Assay

The testes, epididymis, seminal vesicles, and prostate glands were homogenized separately in 50 mM Tris-HCl buffer (pH 7.4) containing 1.15% KCl and the homogenates were centrifuged at 10,000 g for 15 minutes at 4°C. The supernatants were collected for the estimation of CAT activity using hydrogen peroxide as substrate according to the method of Clairborne [13]. SOD activity was determined by measuring the inhibition of autoxidation of epinephrine at pH 10.2 at 30°C according to Misra [14]. Glutathione-S-transferase (GST) activity was estimated by the method of Habig et al. [15] using 1-chloro-2,4-dinitrobenzene (CDNB) as substrate. GPx was assayed by measuring the disappearance of NADPH at 35°C according to Paglia and Valentine [16] and the unit is expressed as moles of NADPH oxidized per milligram of protein. Protein concentration was determined by the method of Lowry et al. [17].

### 2.6. GSH Assay

Reduced GSH was determined at 412 nm using the method described by Jollow et al. [18].

### 2.7. Vitamin C Content Determination

Vitamin C content was determined at 520 nm using the method of Benderitter et al. [19].

### 2.8. Lipid Peroxidation Assay

Lipid peroxidation was quantified as malondialdehyde (MDA) according to the method described by Farombi et al. [20] and expressed as *μ*mole MDA/mg protein.

### 2.9. Statistical Analysis

The data reported herein are the means of five replicates (*n* = 5). Mean separation was done using Fisher's protected least significant difference (LSD) test at *p* < 0.05, *p* < 0.01, *p* < 0.001, and *p* < 0.0001. Linear regression analysis was done to evaluate relationship between control and treated groups. All statistical analyses were done using JMP Release 10.0 statistical package (SAS Institute, Inc., Cary, North Carolina, USA, 2010).

## 3. Results

**Figure 1:**
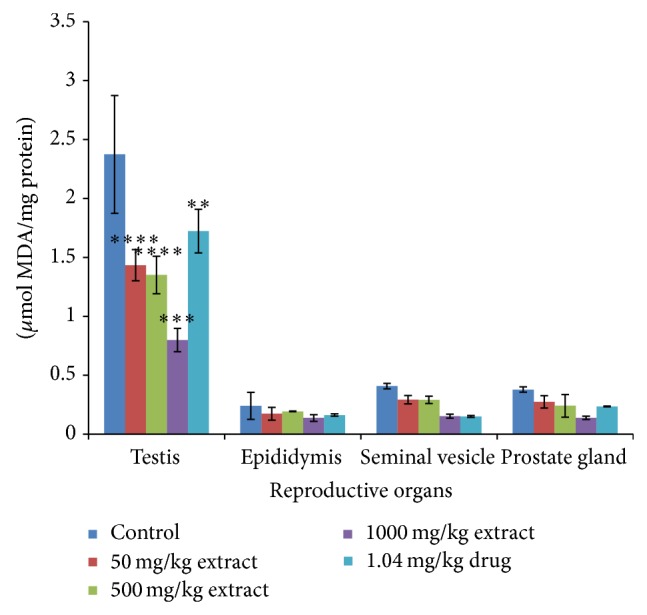
The MDA concentration in the reproductive organs of male rats treated with the* T. conophorum* leaf extract. Data are expressed as mean ± SD, *n* = 5; ^∗^significant (*p* < 0.05), ^∗∗^significant (*p* < 0.01), ^∗∗∗^significant (*p* < 0.001), and ^∗∗∗∗^highly  significant (*p* < 0.0001) compared with the control group.

**Figure 2:**
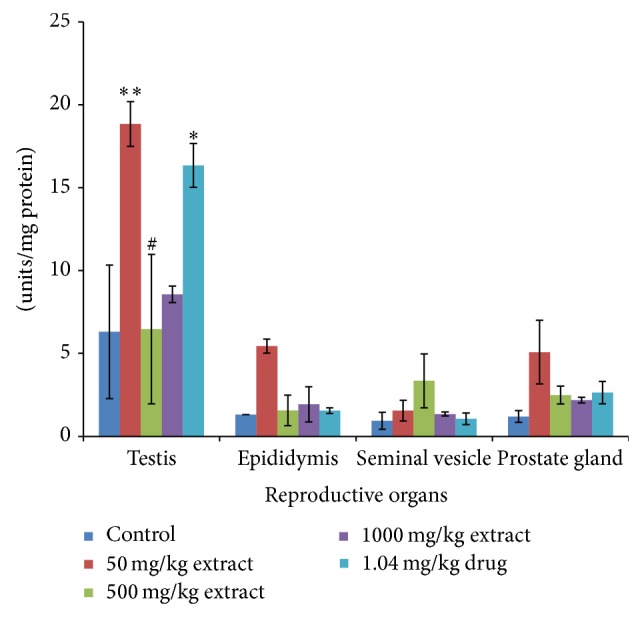
Superoxide dismutase (SOD) levels in the reproductive organs of male rats treated with the* T. conophorum* leaf extract. Data are expressed as mean ± SD, *n* = 5; ^∗^significant (*p* < 0.05), ^∗∗^significant (*p* < 0.01), ^∗∗∗^significant (*p* < 0.001), and ^∗∗∗∗^highly  significant (*p* < 0.0001) compared with the control group, ^#^significant (*p* < 0.05) compared with the standard drug.

**Figure 3:**
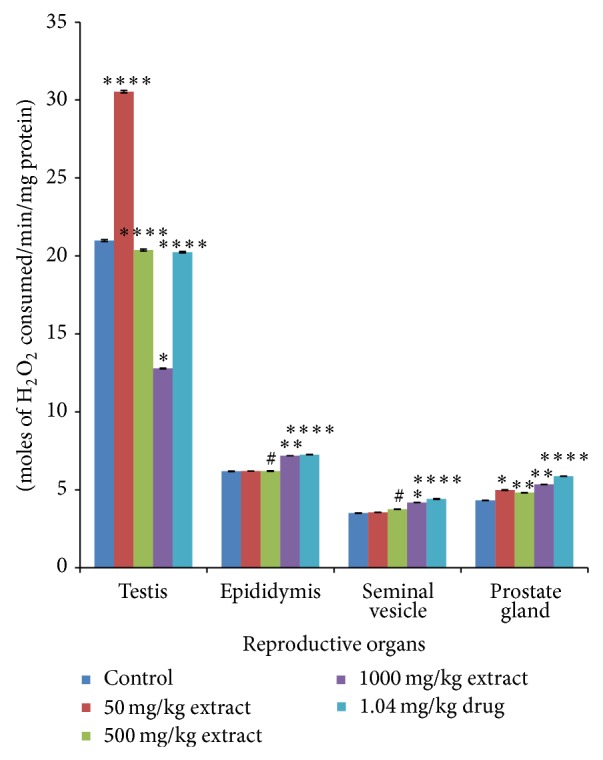
Catalase (CAT) levels in the reproductive organs of male rats treated with the* T. conophorum* leaf extract. Data are expressed as mean ± SD, *n* = 5; ^∗^significant (*p* < 0.05), ^∗∗^significant (*p* < 0.01), ^∗∗∗^significant (*p* < 0.001), and ^∗∗∗∗^highly  significant (*p* < 0.0001) compared with the control group, ^#^significant (*p* < 0.05) compared with the standard drug.

**Figure 4:**
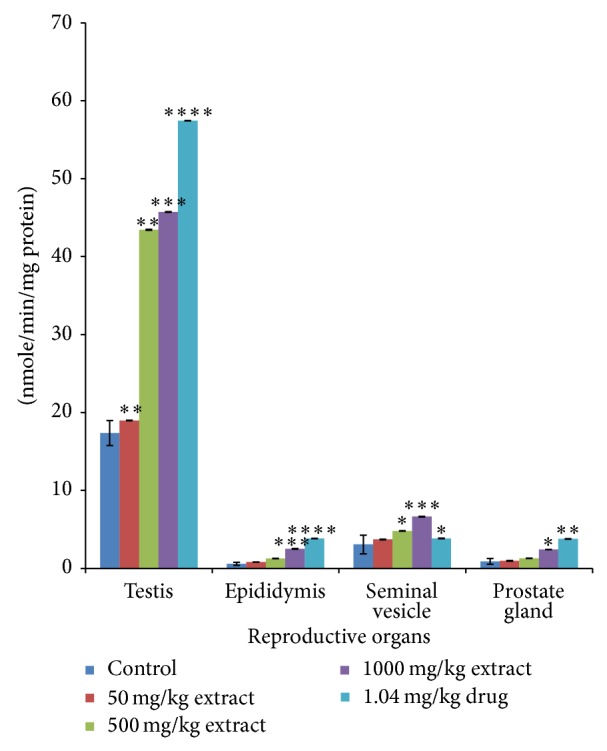
Glutathione-S-transferase (GST) levels in the reproductive organs of male rats treated with the* T. conophorum* leaf extract. Data are expressed as mean ± SD, *n* = 5; ^∗^significant (*p* < 0.05), ^∗∗^significant (*p* < 0.01), ^∗∗∗^significant (*p* < 0.001), and ^∗∗∗∗^highly  significant (*p* < 0.0001) compared with the control group, ^#^significant (*p* < 0.05) compared with the standard drug.

**Figure 5:**
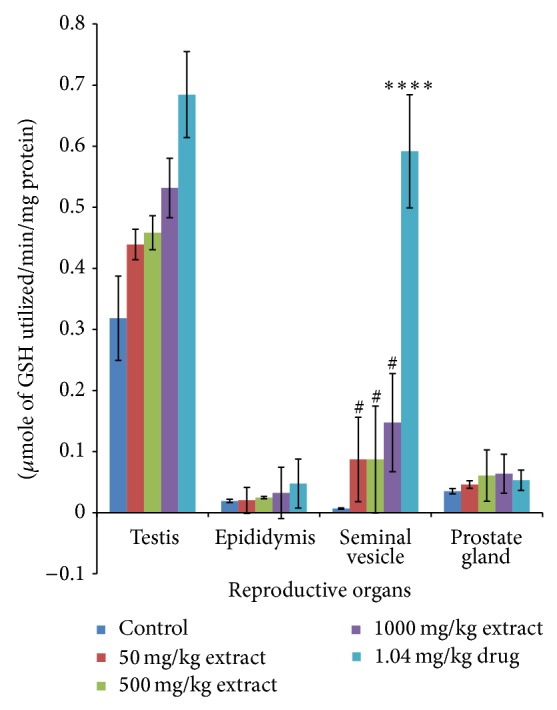
Glutathione peroxidase (GPx) levels in the reproductive organs of male rats treated with the* T. conophorum* leaf extract. Data are expressed as mean ± SD, *n* = 5; ^∗^significant (*p* < 0.05), ^∗∗^significant (*p* < 0.01), ^∗∗∗^significant (*p* < 0.001), and ^∗∗∗∗^highly  significant (*p* < 0.0001) compared with the control group, ^#^significant (*p* < 0.05) compared with the standard drug.

**Figure 6:**
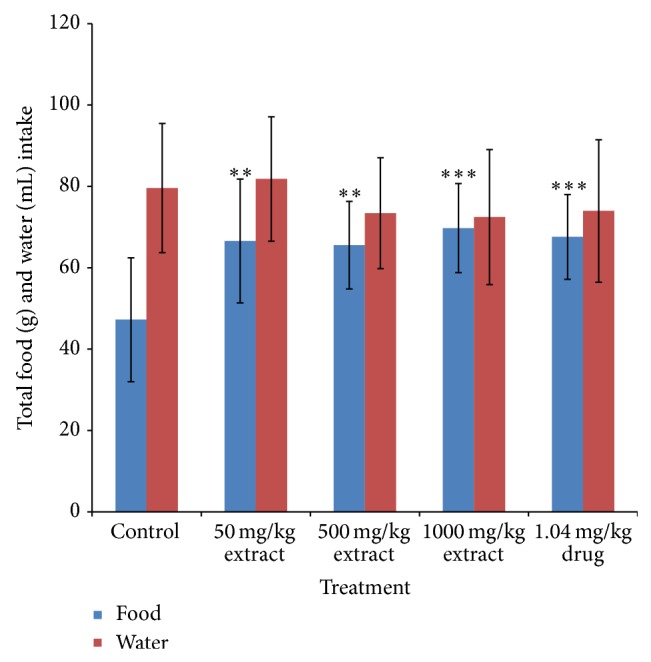
The mean total food and water intake of male albino rats treated with the* T. conophorum* leaf extract. Data are expressed as mean ± SD, *n* = 5; ^∗^significant (*p* < 0.05), ^∗∗^significant (*p* < 0.01), ^∗∗∗^significant (*p* < 0.001), and ^∗∗∗∗^highly  significant (*p* < 0.0001) compared with the control group.

Administration of* T. conophorum* leaf extract caused a significant (*p* < 0.001) and dose-dependent reduction in the lipid peroxidation levels compared with the corresponding group of control animals (Figure 1). The levels of MDA, a maker of lipid peroxidation, in both testis and other accessory glands decreased significantly (*p* < 0.0001) in the extract treated rats in a concentration dependent manner with highest reduction observed at the highest concentration of the extract. At the highest concentration of the extract used the levels of MDA in the testis were reduced significantly about twofold (66.35%) greater than that of the standard drug (27.43%) (Table 1). Other groups also showed highly significant decrease even greater than that of standard drug in the testis. MDA levels were also reduced in the seminal vesicle (28.14, 28.78, and 62.50%) and prostate gland (27.73, 36.26, and 63.36%) at the doses of 50, 500, and 1000 mg/kg, respectively (Table 1), but not significantly different from the control groups. On the other hand, MDA level in the epididymis was very low both in the control and treated groups although there was a nonsignificant reduction at doses 50, 500, and 1000 mg/kg having percentage reduction of 27.58, 19.41, and 42.68%, respectively (Table 1). Additionally, the levels of MDA in the testis decreased more than that in the epididymis, seminal vesicle, and prostate gland compared with the control as shown in Figure 1. The activities of antioxidant enzymes-superoxide dismutase (SOD), catalase (CAT), glutathione peroxidase (GPx), and glutathione-S-transferase (GST) and the levels of GSH, vitamin C, and total protein in the reproductive and accessory organs increased significantly (*p* < 0.05) and in a dose-dependent manner compared with the corresponding group of control animals (Figures 2, 3, 4, and 5, and Table 2). The results of antioxidant enzymes and lipid peroxidation were expressed in terms of milligrams of protein. We also compared the feeding patterns of control group with those groups treated with the extract and fertility drug and we discovered that extract and drug treated groups had normal appetite for food as observed in the significantly higher (*p* < 0.001) food intake and weight gain when compared with control groups (Figure 6, Table 3). Conversely, the group to which extract at 1000 mg/kg and standard drug were administered showed a significant (*p* < 0.05) increase in the testicular weight of rat when compared with control group while the extract and drug significantly increase the weight of the epididymis at all the concentration used. The weights of seminal vesicle and prostate gland slightly increased but were not significantly different from control group (*p* < 0.05) (Table 3).

## 4. Discussion

Although medicinal plants have been shown to contain the bioactive substances with promising antioxidative activities and suspected to be useful to reproductive system by retarding cellular degeneration and preventing cancer caused by free radicals in animals [21–23], our study appears to be the first reporting the antiperoxidative activity of this plant's leaves in reproductive organs and accessory glands of treated rats* in vivo*.

The percentage reduction caused by the extract in all the tissues investigated in this study may be due to the free radical scavenging activities of its phytochemical constituents and these were comparable to that of clomiphene citrate, a fertility drug used as standard in this study. This result was in agreement with the results of previous works where extract of different medicinal plants has been shown to inhibit lipid peroxide formation in several studies [24–29].

Oxidative damage to sperm in many cases of oligospermia coexists with a reduction in the antioxidant capacity, which can increase the deleterious effects of the free radicals [2]. Free radical scavenging enzymes such as SOD, CAT, and glutathione peroxidase (GPx) are the first line of defence against oxidative injury. SOD protects tissues against oxygen free radicals by catalysing the removal of superoxide radical, converting it into H_2_O_2_ and molecular oxygen, which both damage the cell membrane and other biological structures [30]. These radicals (H_2_O_2_ and lipid peroxide) are readily degraded by catalase and glutathione peroxidase using reduced GSH to nontoxic alcohol. Catalase is a haemprotein, which is responsible for the detoxification of significant amounts of H_2_O_2_ [31]. In this study, decreased levels of SOD, CAT, GST, and GPx were observed in normal saline treated group (control) when compared to the extract and fertility drug treated groups. This reduction in untreated rats may be due, in part, to oxidative modification of the enzymatic proteins by excessive ROS generation or may stem from a decrease in their rate of synthesis due to chronic exposure to reactive oxygen insults [31]. We also found that the supplementation with extract and drug significantly caused a marked elevation of SOD, CAT, GST, and GPx, although the extract showed a more pronounced effect than the fertility drug. The lowest concentration of the extract caused the highest increment in SOD and CAT activities in various tissues tested while the extract increased the activities of GST and GPx in a concentration dependent manner. The observed increment in the antioxidant enzymes status in the extract and fertility drug treated groups may be due to enhancement of antioxidant enzyme synthesis by the extract and fertility drug acting on the antioxidant response elements in the enhancer region at the promoter site of the gene that codes for the enzymes [32].

GSH is a major nonprotein thiol and plays a central role in coordinating the antioxidant defence process. It is involved in the maintenance of normal cell structure and function through its redox and detoxification reactions [33]. GSH in association with GST, GPx, and other antioxidant enzymes metabolizes and detoxifies toxic metabolites to less harmful agents before excretion, thereby protecting mammalian cells against oxidative damage. In the present study, we found that the extract caused a highly significant increment in GSH level in all the treated groups compared to control.

A nonsignificant increase was observed in the vitamin C content of various tissues tested in the extract and drug treated groups when compared with control. Also, at 500 and 1000 mg/kg, the extract caused a highly significant increase in total protein level in the seminal vesicle and prostate gland while there was no significant different between the values obtained for other tissues when compared with the control.

Testicular size and weight are the best primary assessment of spermatogenesis. Increases in testicular weight are mostly related to the number of spermatozoa present in the tissue [34]. The significant weight increase of testis in high doses and epididymis in all doses observed in this study directly supports the increase availability of androgen [35]. It is well known that androgen plays an important role in maturation, spermatogenesis, and the maintenance of accessory sex organs [36]. The increase in the testes observed in this study may be due to the increase in the functional ability of the organ while epididymis increment may be due to increasing number of sperm in rat's epididymis which is directly related to the increase of cell population in the seminiferous tubules and increased sperm vitality and motility after extract administration.

## 5. Conclusion

Conclusively, this study clearly demonstrated that the extract showed antiperoxidative activity in rat reproductive organs which was associated with an increase in total protein, nonenzymatic (GSH, vitamin C) and enzymatic (SOD, CAT, GST, and GPx) antioxidant levels under normal condition. This plant may be useful in the treatment/management of reproductive cellular damage involving reactive oxygen species.

## Figures and Tables

**Table 1:** Percentage decrease in the level of MDA produced in the reproductive organs of male rats treated with the *T. conophorum* leaf extract (%).

Treatment groups	Testis	Epididymis	Seminal vesicle	Prostate gland
Control (MDA level)^∗^	2.37 ± 0.50	0.24 ± 0.11	0.41 ± 0.02	0.38 ± 0.02
II	39.60	27.58	28.14	27.73
III	43.09	19.41	28.78	36.26
IV	66.35	42.68	62.50	63.36
V	27.43	32.11	63.04	37.90

^∗^ *μ*mol MDA/mg protein, II, III, and IV: treated with 50, 500, and 1000 mg/kg. BW/day of extract, V: treated with 1.04 mg/kg. BW/day of clomiphene citrate.

**Table 2:** Total protein (TPC), reduced glutathione (GSH), and vitamin C contents in the reproductive organs of male rats treated with the *T. conophorum* leaf extract.

Variables	Control	50 mg/kg extract	500 mg/kg extract	1000 mg/kg extract	1.04 mg/kg drug
Testis					
TPC^a^	6.66 ± 2.89	10.00 ± 1.25^∗^	10.00 ± 1.25^∗^	15.83 ± 3.15^∗^	15.00 ± 0.00^∗^
GSH^b^	24.94 ± 0.09	59.25 ± 0.29^∗∗∗∗^	39.31 ± 0.51^∗∗∗∗^	25.02 ± 0.00	38.78 ± 0.59^∗∗∗∗^
Vitamin C^c^	2.19 ± 0.09	2.25 ± 0.99	3.08 ± 0.14	3.26 ± 0.03	3.10 ± 0.81
Epididymis					
TPC^a^	117.71 ± 10.00	118.64 ± 18.00	120.08 ± 10.00	135 ± 19.00^∗^	121.24 ± 25.00^∗^
GSH^b^	11.48 ± 0.02	13.33 ± 0.01^∗∗∗∗^	14.68 ± 0.03^∗∗∗∗^	15.77 ± 1.00^∗∗∗∗^	14.77 ± 0.04^∗∗∗∗^
Vitamin C^c^	2.45 ± 0.01	2.49 ± 0.07	2.52 ± 0.12	2.67 ± 0.10	2.71 ± 0.09
Seminal vesicle					
TPC^a^	54.17 ± 5.90	57.25 ± 8.00^#^	58.33 ± 3.14	71.83 ± 12.64^∗∗∗∗^	63.42 ± 2.03
GSH^b^	6.44 ± 0.08	6.72 ± 0.00	7.10 ± 0.06	9.92 ± 0.01^∗∗∗∗^	11.14 ± 0.04^∗∗∗∗^
Vitamin C^c^	1.58 ± 0.06	1.59 ± 0.04	1.68 ± 0.05	1.70 ± 0.03	1.73 ± 0.02
Prostate gland					
TPC^a^	61.25 ± 1.25	67.50 ± 2.16	75.67 ± 7.21^∗∗∗∗^	81.67 ± 4.73^∗∗∗∗^	70.33 ± 4.64
GSH^b^	10.35 ± 0.06	11.97 ± 0.01	14.51 ± 0.09^∗∗^	20.44 ± 0.13^∗∗∗∗^	16.17 ± 0.20^∗∗∗∗^
Vitamin C^c^	1.49 ± 0.61	1.51 ± 0.59	1.53 ± 0.74	1.55 ± 0.62	1.57 ± 0.58

^a^mg/mL, ^b^ *µ*mole of GSH/mg protein, and ^c^mmol/mg protein. Data are expressed as mean ± SD, *n* = 5. ^∗^significant (*p* < 0.05), ^∗∗^significant (*p* < 0.01), ^∗∗∗^significant (*p* < 0.001), and ^∗∗∗∗^highly significant (*p* < 0.0001) compared with the control group, ^#^significant (*p* < 0.05) compared with the standard drug.

**Table 3:** Body and reproductive organ weights of male albino rats treated with the *T. conophorum* leaf extract.

Parameters	Control	50 mg/kg extract	500 mg/kg extract	1000 mg/kg extract	1.04 mg/kg drug
Initial body weight (g)	348 ± 7.81	307 ± 15.82	305 ± 28.93	309 ± 24.41	317 ± 7.81
Final body weight (g)	368 ± 12.42	339 ± 14.19	342 ± 14.53	345 ± 13.47	295 ± 12.41
Weight gain (%)	20	32^∗^	37^∗^	36^∗^	22
Right testis					
Absolute weight (g)	1.88 ± 0.04	1.88 ± 0.11	1.95 ± 0.11	2.95 ± 0.11^∗^	3.06 ± 0.18^∗^
Weight (g/100 g BW)	0.63 ± 0.01	0.64 ± 0.03	0.65 ± 0.04	0.75 ± 0.04	0.69 ± 0.06
Left testis					
Absolute weight (g)	2.11 ± 0.05	2.16 ± 0.02	2.51 ± 0.08	3.04 ± 0.45^∗^	3.11 ± 0.06^∗^
Weight (g/100 g BW)	0.67 ± 0.00	0.67 ± 0.01	0.68 ± 0.02	0.62 ± 0.01	0.66 ± 0.03
Epididymis					
Absolute weight (g)	0.47 ± 0.05	1.42 ± 0.11^∗^	1.46 ± 0.11^∗^	1.48 ± 0.12^∗^	1.49 ± 0.20^∗^
Weight (g/100 g BW)	0.50 ± 0.03	0.44 ± 0.03	0.46 ± 0.03	0.49 ± 0.04	0.51 ± 0.06
Seminal vesicle					
Absolute weight (g)	0.77 ± 0.15	0.84 ± 0.12	0.93 ± 0.04	1.09 ± 0.41	0.94 ± 0.10
Weight (g/100 g BW)	0.46 ± 0.03	0.24 ± 0.02	0.25 ± 0.03	0.26 ± 0.11	0.26 ± 0.03
Prostate gland					
Absolute weight (g)	1.00 ± 0.10	1.07 ± 0.18	1.18 ± 0.28	1.20 ± 0.22	1.19 ± 0.01
Weight (g/100 g BW)	0.60 ± 0.00	0.32 ± 0.05	0.32 ± 0.07	0.35 ± 0.07	0.36 ± 0.08

Data are expressed as mean ± SD, *n* = 5. ^∗^Significant (*p* < 0.05), ^∗∗^significant (*p* < 0.01), ^∗∗∗^significant (*p* < 0.001), and ^∗∗∗∗^highly significant (*p* < 0.0001) compared with the control group, ^#^significant (*p* < 0.05) compared with the standard drug.
